# Unexpected polymorphism during a catalyzed mechanochemical Knoevenagel condensation

**DOI:** 10.3762/bjoc.15.110

**Published:** 2019-05-21

**Authors:** Sebastian Haferkamp, Andrea Paul, Adam A L Michalchuk, Franziska Emmerling

**Affiliations:** 1 BAM Federal Institute for Materials Research and Testing, Richard-Willstaetter-Straße 11, 12489 Berlin, Germany; 2 Humboldt-Universität zu Berlin, Brook-Taylor-Straße 2, 12489 Berlin, Germany

**Keywords:** ball milling, C–C coupling, in situ, mechanochemistry, multivariate data analysis

## Abstract

The transformation of a base-catalyzed, mechano-assisted Knoevenagel condensation of mono-fluorinated benzaldehyde derivatives (*p*-, *m*-, *o*-benzaldehyde) with malonodinitrile was investigated in situ and in real time. Upon milling, the *para*-substituted product was found to crystallize initially into two different polymorphic forms, depending on the quantity of catalyst used. For low catalyst concentrations, a mechanically metastable phase (monoclinic) was initially formed, converting to the mechanically stable phase (triclinic) upon further grinding. Instead, higher catalyst concentrations crystallize directly as the triclinic product. Inclusion of catalyst in the final product, as evidenced by mass spectrometric analysis, suggests this complex polymorphic pathway may be due to seeding effects. Multivariate analysis for the in situ Raman spectra supports this complex formation pathway, and offers a new approach to monitoring multi-phase reactions during ball milling.

## Introduction

Mechanochemistry offers a wide array of applications. It is used widely for synthesis of inorganic, metal-organic, and organic molecules and materials [1]. Interest in these methods stems largely from the fact that they are efficient and more environmentally friendly as compared to traditional approaches [2–3]. Mechanochemistry is a well-established method for the synthesis of coordination polymers, the formation of cocrystals, and in C–C coupling reactions [4–7].

Despite the increasing use of mechanochemistry, there is still a lack in understanding of the underlying processes involved during mechanically-facilitated reactions. This is particularly true of the potential role of transient polymorphic phases [8] and seeding effects [9] in understanding reaction kinetics of these processes. Early insight into formation pathways was provided ex situ, in which the mechanical treatment was stopped, and powder removed for analysis [10–11]. More recently, further detail has been gained by monitoring mechanochemical transformations in real time, using in situ techniques [12–13]. The first in situ and real time study was performed by X-ray powder diffraction (XRPD) to monitor transformations during a milling process [12]. In situ studies allow novel insight into the mechanism of a mechanochemical process, without changing the reaction environment. It was subsequently demonstrated how a combination of different in situ methods can provide more thorough investigation of mechanochemical reaction mechanisms [14–16]. Of particular benefit to synthetic reactions, such as C–C bond formation [17–18], the use of Raman spectroscopy is of great interest. The characteristic bands are usually well separated, and the course of the reaction can be followed easily. The advantage of Raman spectroscopy was recently demonstrated [19], where its combination with XRPD allowed monitoring of the mechanochemically catalyzed Knoevenagel condensation in detail.

## Results and Discussion

**Scheme 1:**
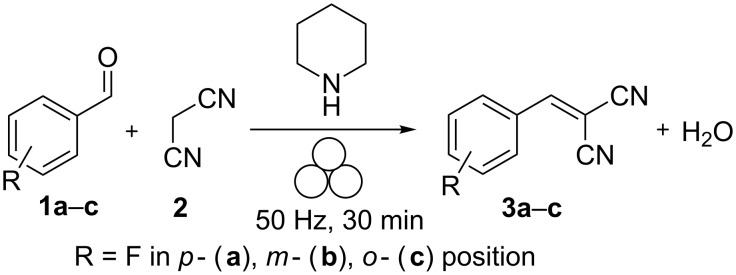
Catalyzed mechanochemical Knoevenagel condensation of fluorobenzaldehydes and malonodinitrile. The milling process is symbolized by the three balls, proposed by Hanusa et al. [20].

**Figure 1:**
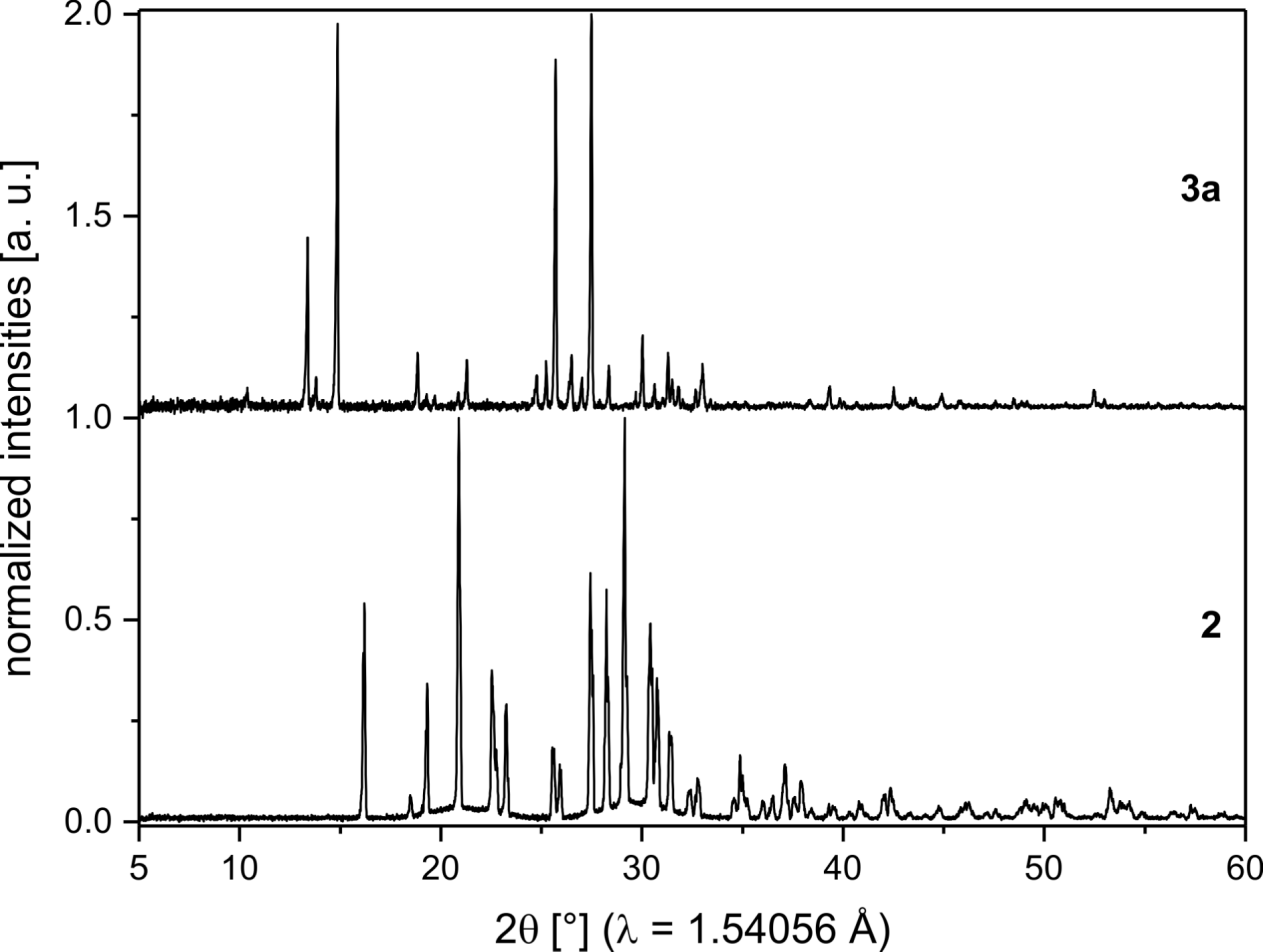
Comparison of XRPD pattern of malonodinitrile (**2**) and (*p*-fluorobenzylidene) malonodinitrile (**3a**). The patterns are baseline corrected.

The catalyzed Knoevenagel condensations of mono-fluorinated benzaldehydes **1a**–**c** with malonodinitrile (**2**) are depicted in Scheme 1. In contrast to previous work, which reported the uncatalyzed reaction [19], piperidine was used as a basic catalyst. This was done as the inclusion of the base led to crystallization of the corresponding products **3a**–**c** during the milling process. In contrast, crystallization during the base-free reaction required an ex situ aging step. Figure 1 shows the XRPD pattern of the substrate **2** and the product **3a**. Due to the liquid state of the fluorinated benzaldehydes **1a**–**c** no XRPD pattern could be recorded.

**Figure 2:**
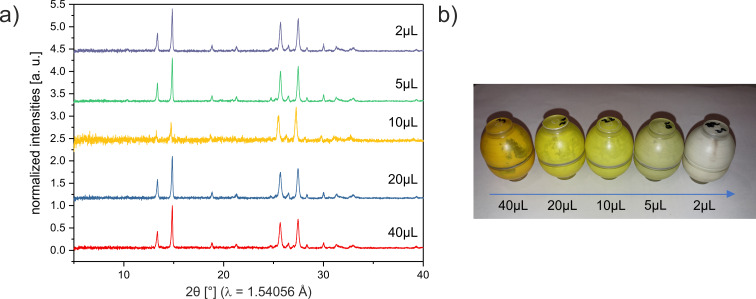
a) XRPD pattern of (*p*-fluorobenzylidene)malonodinitrile (**3a**) direct after the synthesis with different amounts of catalyst. b) Color change of the product **3a** after the synthesis with different amounts of catalyst. The amount is reduced in the direction of the arrow.

**Figure 3:**
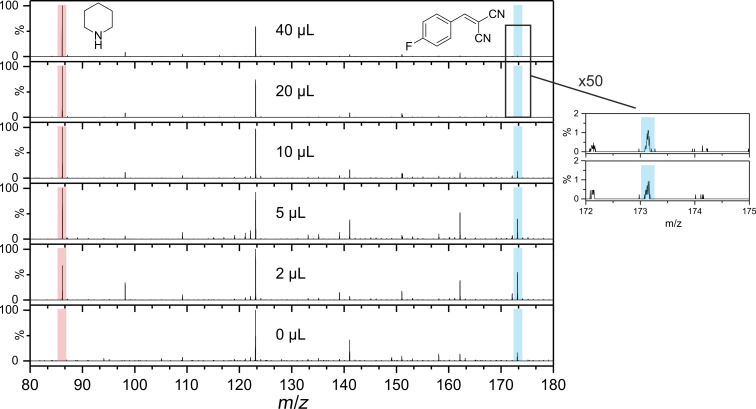
Mass spectra of the different products of **3a**. Red: peak off the molecular ion [M + H]^+^ of piperidine (*m*/*z* ≈ 86). Blue: peak of the molecular ion [M + H]^+^ of **3a** (*m*/*z* ≈ 173).

For the reaction of **1a** with **2**, the amount of catalyst was varied and the reaction monitored in each case. XRPD analysis confirmed that, independent of the amount of catalyst, the same bulk product was formed. The intensity of the product powder color decreased from a deep orange to white, with decreasing quantity of catalyst (Figure 2b). Importantly, none of the starting materials are colored, and the color of the product phase was found to vary systematically with increasing catalyst concentration. This coloring was observed throughout the bulk, which therefore suggested that the catalyst was incorporated into the structure of the solid product. Such effects have been previously reported, and have the potential to seed nucleation of polymorphic phases [21]. For that reason, mass spectrometric analyses of the powder phases were performed (Figure 3). The relative intensity of the peak corresponding to piperidine (Figure 3 red box) decreases systematically with respect to that of **3a** (Figure 3 blue box). Hence, this suggests that the catalyst is indeed present in the solid product. Further analysis is required to understand the nature of catalyst incorporation, and hence the origin of color.

**Figure 4:**
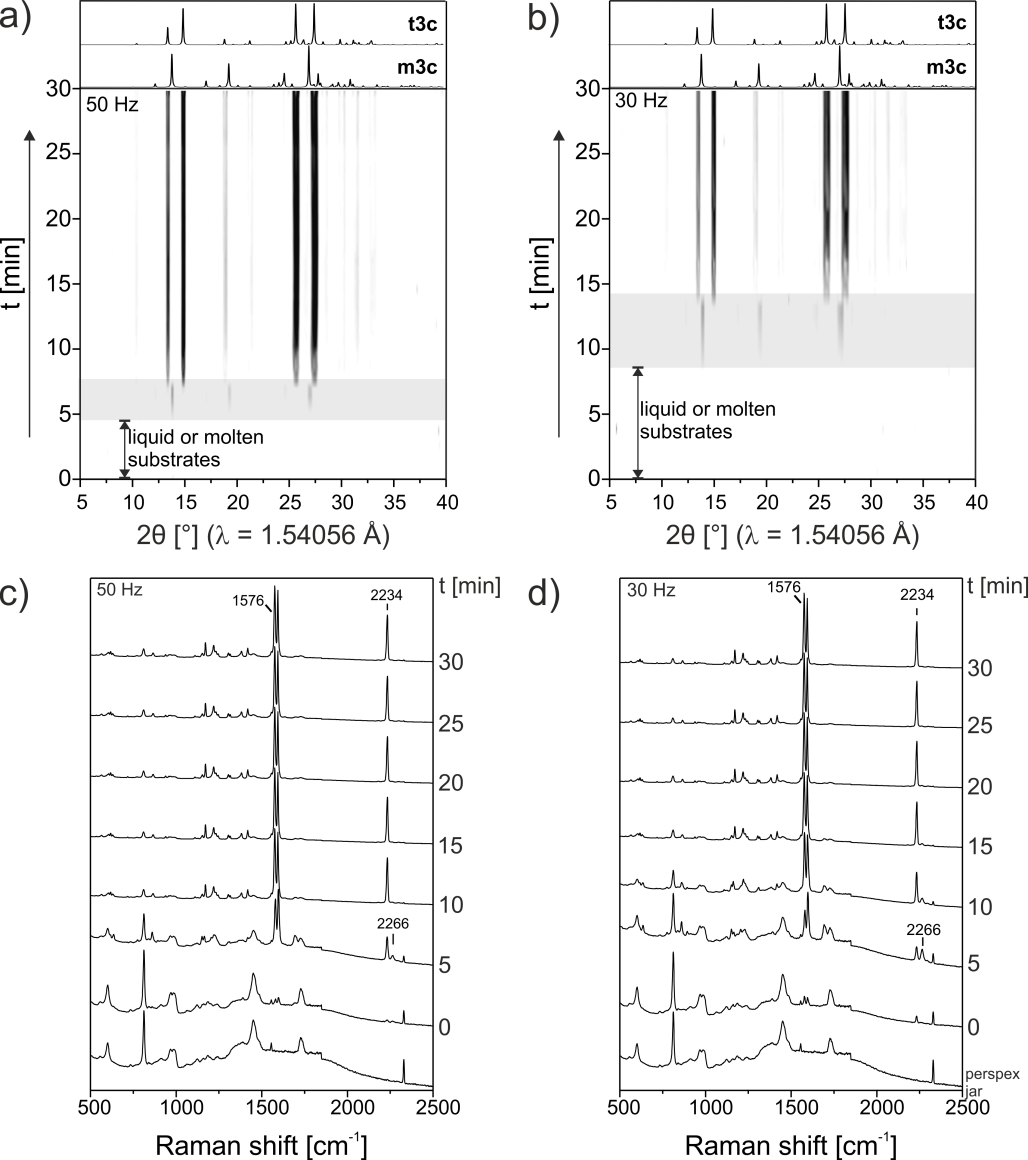
a) In situ XRPD pattern of the mechanochemical Knoevenagel condensation of **1a** and **2** with 2 µL catalyst at 50 Hz. Gray box: intermediate phase. b) In situ XRPD pattern of the reaction with a milling frequency of 30 Hz. c) In situ Raman spectra of the mechanochemical Knoevenagel condensation of **1a** and **2** with 2 µL catalyst at 50 Hz. d) In situ Raman spectra of the reaction with a milling frequency of 30 Hz. Raman bands: 1576 cm^−1^ – C-C stretching vibration; 2234 cm^−1^ – C≡N stretching vibration of the product; 2266 cm^−1^ – C≡N stretching vibration of malonodinitrile.

The reactions containing between 40 µL and 5 µL catalyst show conversion of the substrates directly into the mechanically stable triclinic product phase, according to real-time in situ XRPD analysis (Supporting Information File 1, Figure S1). Consistent with previous reports [15], **2** melted at the beginning of the reaction and remained liquid or molten for the first few minutes of milling (Supporting Information File 1). This is due to increased temperatures within the jar during milling (*T*_melt_(**2**) = 32 °C). Shortly after melting of **2,** Bragg reflections of a crystalline product phase were found to form (Figure 4). Comparison to literature crystallographic data suggests this to be the triclinic phase (**t3a**). This phase remained stable upon continued milling.

In contrast, reactions conducted with 2 µL were found to be more complex (Figure 4). Shortly after the melting of **2**, Bragg reflections were observed and remained visible for a period of approximately two minutes (Figure 4, gray box). These reflections, however, were found to correspond to the monoclinic phase of the product (**m3a**) [22–23]. Phase **m3a** remained stable under mechanical treatment for a few minutes, before transforming abruptly (over a period of 90 seconds) to the **t3a** phase. Having observed the inclusion of catalyst into the final product phase (see Figure 3), we suggest this change in polymorphic behavior to result from a templating phenomenon, which dominates at higher concentrations of catalyst.

**Figure 5:**
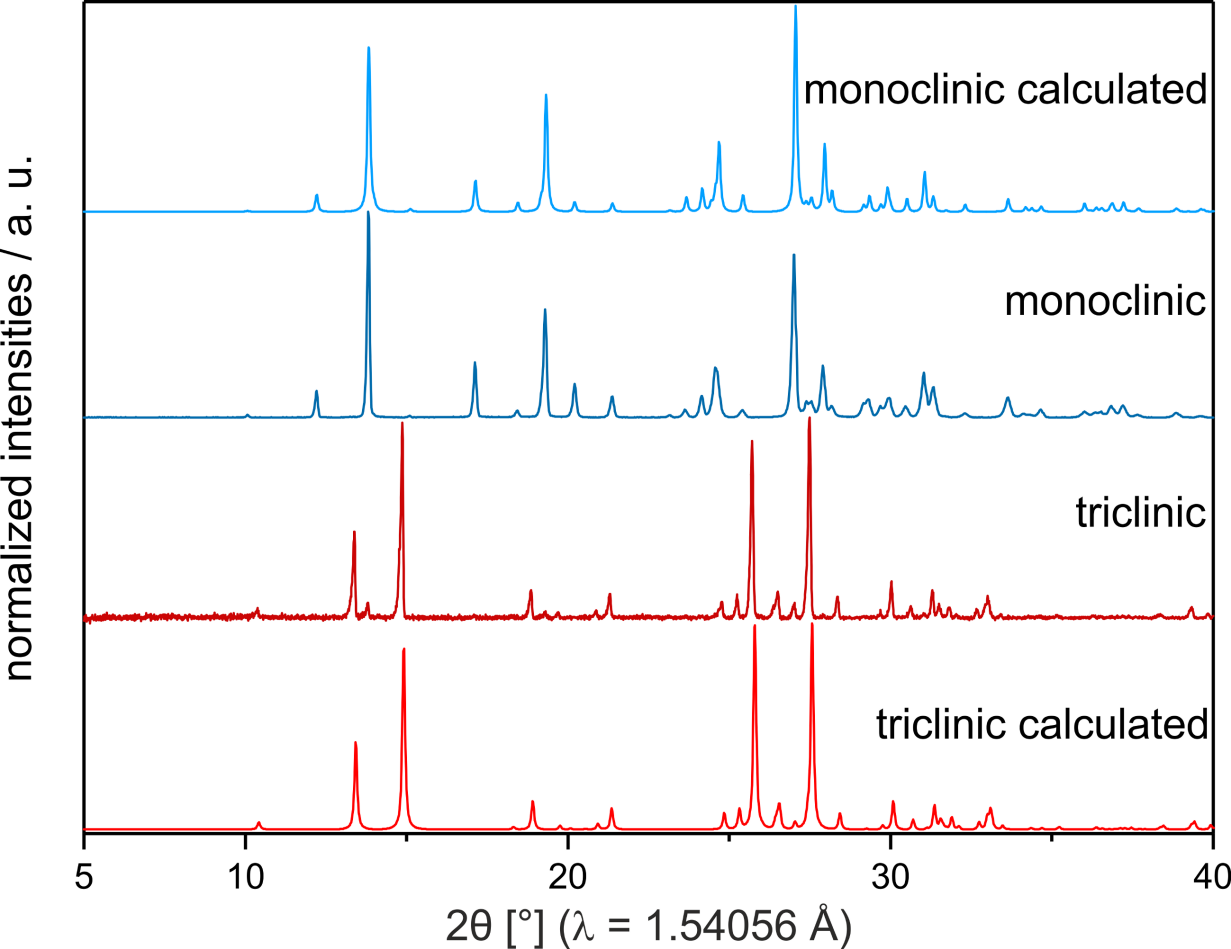
Comparison of XRPD patterns of both polymorphs of the product **3a**. Red: triclinic polymorph **t3a**. Blue: monoclinic polymorph **m3a**. The light XRPD pattern were calculated from single crystal diffraction data.

To better observe this transformation pathway, the reaction (2 µL catalyst) was repeated at 30 Hz in order to extend the lifetime of **m3a**. The in situ XRPD pattern and Raman spectra are shown in Figure 4b and d, respectively. Decreasing the milling frequency has a number of notable effects. First, the lifetime of the initial molten/liquid phase was nearly doubled, suggesting that mechanical treatment has an important effect on this largely solution-phase reaction. This may be a result of heating, mechanical activation of the fluid-phase molecules or differences in energy for nucleation. While further work is required in this area, it is clear that mechanical treatment can have notable influence on ‘solution-phase’ chemistry. Second, the lifetime of the intermediate phase was extended. This allowed us to collect higher resolution XRPD data by extracting a sample from the milling vessel and confirming this intermediate to be the phase, **m3a** (Figure 5). Despite this extension, however, the subsequent transformation **m3a → t3a** again occurs abruptly (ca. 120 seconds). This suggests that the transformation may result from the accumulation of defects within **m3a**, or upon comminution of the product phase [24].

While the XRPD patterns clearly show the occurrence of a transition phase within the first minutes of the reaction, the Raman spectra represent a superposition of all spectral components. These spectral features often remain unchanged for polymorphic series. For this reason, it is difficult to employ reliably Raman spectroscopy for in situ mechanochemical data. Hence, despite its availability in many laboratories, in situ real-time monitoring of mechanochemistry by Raman scattering has remained underexplored. We therefore sought to identify a means to resolve this issue, using multivariate methods to analyze the reaction. Principal component analysis (PCA) is a bilinear modelling method [25–26] that helps to extract the main information from multi-dimensional data (here a time series of Raman spectra). The information contained within the original spectral variables is projected onto a small number of underlying (‘latent’) variables, called the principal components (PC). Typically, the first PC covers the highest variance, the second and following PCs cover less information. All PCs are orthogonal to one-another. Although the PCs themselves do not represent quantitative data, they represent the underlying chemical or physical processes. In order to understand the results of PCA, both scores and loadings must be scrutinized in parallel. Whereas the scores contain information regarding the samples, the loadings provide information on the variables. High loading values indicate spectral variables of high importance. The same applies for large negative values.

**Figure 6:**
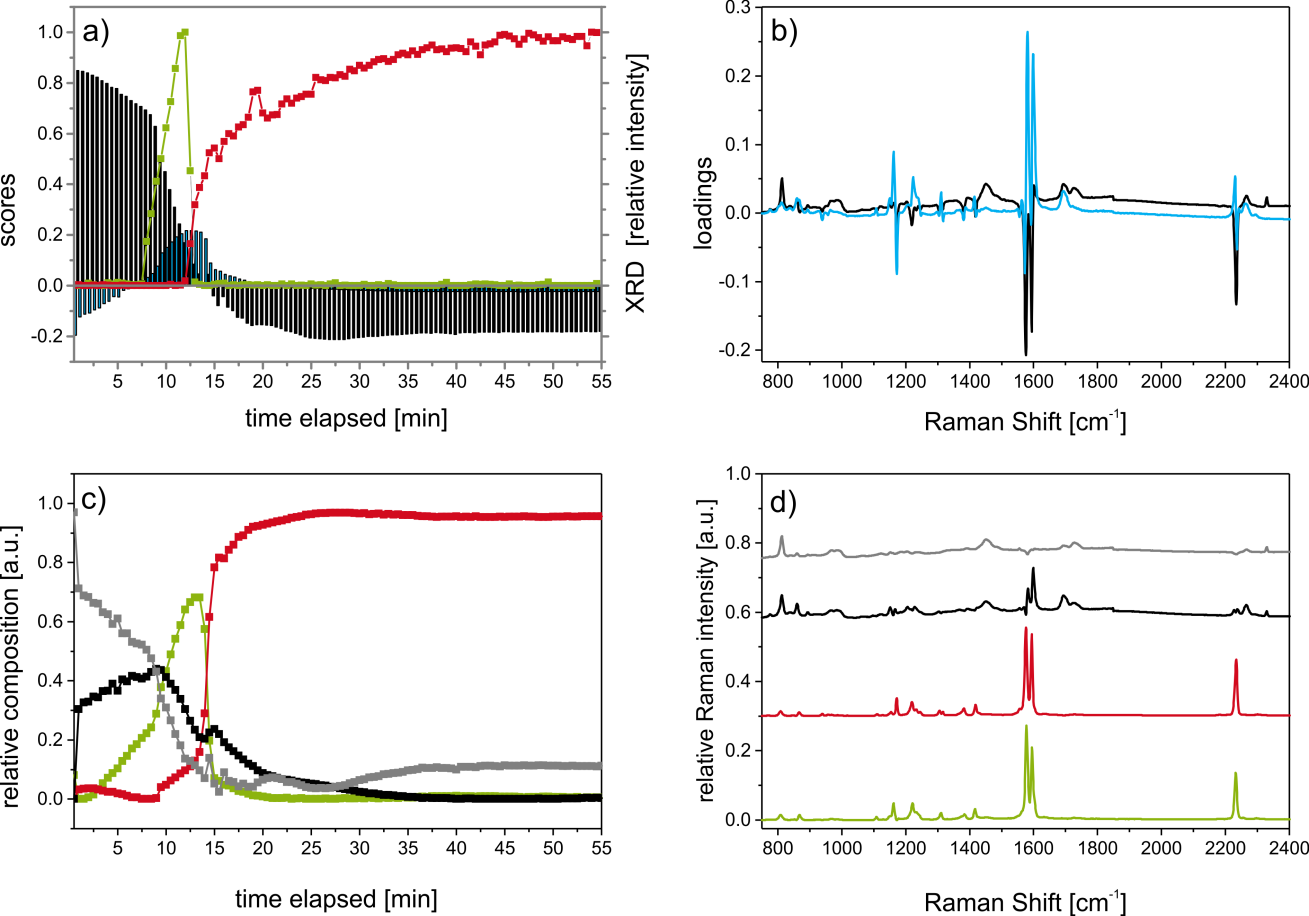
Results of multivariate data analysis of Raman spectra for 30 Hz milling experiments. Principal component analysis (PCA) of Raman data, showing (a) scores, and (b) loadings for (black) PC1 and (blue) PC2. (a) PC1 scores show a decrease in spectral component of **1a** in the first minutes – cf. positive spectral signature of loadings of PC1 in (b). The negative scores of PC1 correspond to the negative part of PC1 loadings, i.e., **t3c**. Scores of PC2 indicate the presence of a compound with spectral features resembling **m3c**. For comparison, intensity of PXRD Bragg peaks at (green) 2θ = 13.87° and (red) 2θ = 14.78° are given in (a) for **m3c** and **t3c,** respectively. Multivariate curve resolution (MCR) of (c) composition profiles, and (d) component spectra. Coloring of (c) and (d) are used to indicate corresponding data. (c) Relative phase composition of (black) **1a** and the (grey) PMMA-background decrease within the first minutes of the reaction. Simultaneous formation of (green) **m3c**, shows pronounced increase followed immediately by a rapid decrease. The latter is accompanied by the formation of (red) **t3c**. In the final stage of the reaction, only contributions from **t3c** and the PMMA background can be observed.

PCA of the in situ Raman spectra in this work reveals two principal components (PC) for both milling reactions (Supporting Information File 1, Figure S2a–d). The score and loading plots for the first (PC1) and second (PC2) principal component depicted in Figure 6a,b refer to the reaction performed at 30 Hz. PC1 and PC2 account for 96% and 3% of the spectral variance. The scores of PC1 exhibit high values at the beginning of the reaction. With increased milling time, the PC1 scores decline and eventually become negative, indicating the conversion of **1a** to **3a**. Concerning PC1 in Figure 6b, the positive part corresponds to spectral features of **1a** and PMMA, and the negative part indicates features of **3a**. In contrast, the scores of PC2 are initially negative, rise to a peak value, and subsequently fall to zero (cf. Figure 6a). The spectral loadings of PC2 in Figure 6b show a dominant positive structure, which resembles the negative profile of PC1, albeit shifted slightly towards larger wavenumbers. We therefore conclude that PC2 represents an independent (orthogonality among principal components) transient species. A comparison with the Raman spectra summarized in Figure S2 indicates that PC2 correlates well with the formation of **m3a**. Identical results were found for the PCA of in situ Raman spectra at 50 Hz milling frequency (cf. Supporting Information File 1, Figure S2). However, the reduction of the milling frequency delays the onset of product formation. The kinetic profile obtained by PCA correlates well with XRPD data (cf. Figure 6a), in which the transformation is monitored by the normalized intensities of Bragg reflections at 2θ = 13.87° and 14.78°. While the XRPD patterns indicate formation of two clearly separated compounds, the Raman spectra suggest the parallel presence of **1a, m3a** and **t3a**.

Multivariate curve resolution (MCR) aims to extract information on the pure compounds [27]. In our case, the time series of Raman spectra may be considered as a superposition of all compounds present at a certain time of the reaction. In contrast to PCA, where only the variance of data is evaluated, in MCR chemical knowledge on the number of involved compounds, some constraints such as non-negativity are included. As a result, relative component concentrations as a function of time and the corresponding spectra can be evaluated. In this case, four spectral compounds were used in form of an initial guess for MCR, namely fluorinated benzaldehyde, PMMA (background from the milling jar), monoclinic and triclinic fluorobenzylidene malonodinitrile.

MCR analysis without an initial guess resulted in models with three and four components, each containing mixed information from more than one chemical compound (not shown). However, MCR using spectra from the pure materials for an “initial guess” leads to component profiles for **m3a** and **t3a** (Figure 6c) that are in good agreement with XRPD results (Figure 6a and c). The component spectra obtained by MCR can be assigned to the **m3a, t3a, 1a,** and the milling jar, PMMA (Figure 6d, Figure S3, Supporting Information File 1). Negligible contributions of spectral features relating to (**2**) were observed. It should be noted that all component spectra, especially those of (**1a**) contain spectral properties belonging to PMMA (shaded areas in Figure S3, Supporting Information File 1).

The mechanochemical catalyzed reactions of **1b** and **1c** with **2** performed with 2 µL piperidine did not exhibit the same polymorphic transformations as with **1a.** Instead, reactions using **1b** and **1c** led directly to formation of **m3b** [23] and **m3c** [28], respectively, at both 30 Hz and 50 Hz milling frequency (Figures S4 and S5 in Supporting Information File 1).

## Conclusion

The mechano-assisted catalyzed Knoevenagel condensation of mono-fluorinated benzaldehydes and malonodinitrile was explored in situ and in real-time by tandem synchrotron powder X-ray diffraction and Raman spectroscopy. For synthesis of *p*-fluorobenzylidene malonodinitrile (**3a**) the reaction product crystallizes according to two different pathways, depending on the concentration of base catalyst. At high concentrations of catalyst, the triclinic product phase is formed, and remains stable under continued mechanical treatment. In contrast, at lower concentrations of catalyst, the product crystallizes first as the monoclinic phase. Subsequent milling causes this phase to transform abruptly to the triclinic phase. Due to the inclusion of base catalyst in the final product, we suggest this difference to be the result of a templating effect, which dominates at higher concentrations. For the reaction of *meta-* and *ortho-*substituted substrates, crystallization occurs directly into the monoclinic phase, regardless of milling conditions or catalyst concentration.

Multivariate analysis of in situ Raman spectra by both PCA and MCR suggests the formation of a transient product with almost identical spectral properties as the final product, the triclinic polymorph of **3a**. These results are consistent with those of XRPD analysis. Hence, we here identify a new approach to monitoring mechanically-induced polymorphic transitions in situ and in real-time.

## Experimental

**Materials:** All chemicals used in this work were taken as supplied (>97% purity), without further purification.

**Syntheses:** The following procedure is similar as described in our previous work [29]. Milling experiments were performed using a commercially available vibratory ball mill (Pulverisette 23, Fritsch, Germany). For each experiment, stoichiometric quantities of reactants *p*-, *m*- and *o*- fluorobenzaldehyde (500 mg, ca*.* 4.03 mmol) and malonodinitrile (266.1 mg, ca. 4.03 mmol) were weighed into Perspex milling jars (10 mL). To each jar, a quantity (defined in the main text) of piperidine was added as catalyst. Two stainless steel milling balls (4 g, 10 mm diameter) were also included in each milling jar. The reactions were conducted at 30 Hz or 50 Hz, as indicated in the main text. The final products were characterized by XRPD.

**X-ray powder diffraction (XRPD):** All samples were characterized by XRPD analysis using a Bruker D8 diffractometer with Cu-Kα_1_ radiation (λ = 1.54106 Å) in a range of 5.0° ≤ 2 θ ≤ 40°. The data were obtained in transmission mode with a step size of 0.009° and an acquisition time of 3 s per step.

**In situ investigations:** In situ and real-time monitoring of the milling reactions was conducted at the mySpot Beamline (BESSY II, Helmholtz Centre Berlin for Materials and Energy). The same mechanochemical reactor was used for these investigations, as was used for laboratory synthesis reactions; i.e., a Pulverisette 23, Fritsch, Germany. Perspex milling jars were used, which have been previously shown to permit collection of good quality XRPD data during milling reactions [14]. Diffraction was collected using an incident beam of 12.4 keV. 2D scattering images were recorded on a MarMosaic, CCD detector (resolution 3072 × 3072 pixel). All scattering data were processed using FIT2D [30]. In situ real-time Raman data were collected using a non-contact probe (beam diameter 1 mm) and excitation wavelength of 785 nm. Raman scattering was collected on a RXN1^TM^ analyzer (Kaiser Optical systems, France), equipped with a CCD detector (1024 × 1024 pixel). Each Raman spectrum consists of 5 s accumulated scattering intensity, with successive Raman spectra collected every 30 s.

**Chemometrics:** The Raman spectra were evaluated using principle component analysis (PCA) and multivariate curve resolution (MCR) with the software The Unscrambler^®^ X Vers. 10.5 (CAMO). Prior to multivariate analysis, Raman spectra were baseline corrected followed by unit vector normalization in the spectral range of 200–2500 cm^−1^. PCA was conducted with mean centered data using cross validation with 20 randomly selected segments. MCR iterations were initialized with the constraints of "non-negative spectra" and "non-negative concentrations", and sensitivity to pure compounds was set to 100. The maximum number of iterations was set at 250.

**Mass spectrometry:** Mass spectra were recorded with electrospray ionization time of flight mass spectrometry. A Q-TOF Ultima ESI-TOF mass spectrometer (Micromass, Germany) running at 4 kV capillary voltage and a cone voltage of 35 V was used. The collision energy was set to 5 eV. The source temperature was 120 °C whereas the desolvation temperature was adjusted to 150 °C. The mass spectrometer was operating in positive ion mode. Around 0.1 mg of the samples were weigh-in and solved in methanol (HPLC grade).

## Supporting Information

File 1 XRPD data and multivariate data analysis.
