# Supplementary material for: Unexpected polymorphism during a catalyzed mechanochemical Knoevenagel condensation
Source: Beilstein J Org Chem. 2019 May 21;15:1141–8. doi: 10.3762/bjoc.15.110 (PMC6541349; doi:10.3762/bjoc.15.110)
Supplement: File 1 — XRPD data and multivariate data analysis. [file Beilstein_J_Org_Chem-15-1141-s001.pdf]

BEILSTEIN JOURNAL OF ORGANIC CHEMISTRY

Supporting Information

for

Unexpected polymorphism during a catalyzed mechanochemical Knoevenagel condensation

Sebastian Haferkamp, Andrea Paul, Adam A. L. Michalchuk and Franziska Emmerling

Beilstein J. Org. Chem. **2019,** *15,* 1141–1148. doi:10.3762/bjoc.15.110

XRPD data and multivariate data analysis

License and Terms: This is a supporting information file under the terms of the Creative Commons Attribution License (http://creativecommons.org/licenses/by/4.0). Please note that the reuse, redistribution and reproduction in particular requires that the authors and source are credited.
The license is subject to the *Beilstein Journal of Organic Chemistry* terms and conditions: (https://www.beilstein-journals.org/bjoc)

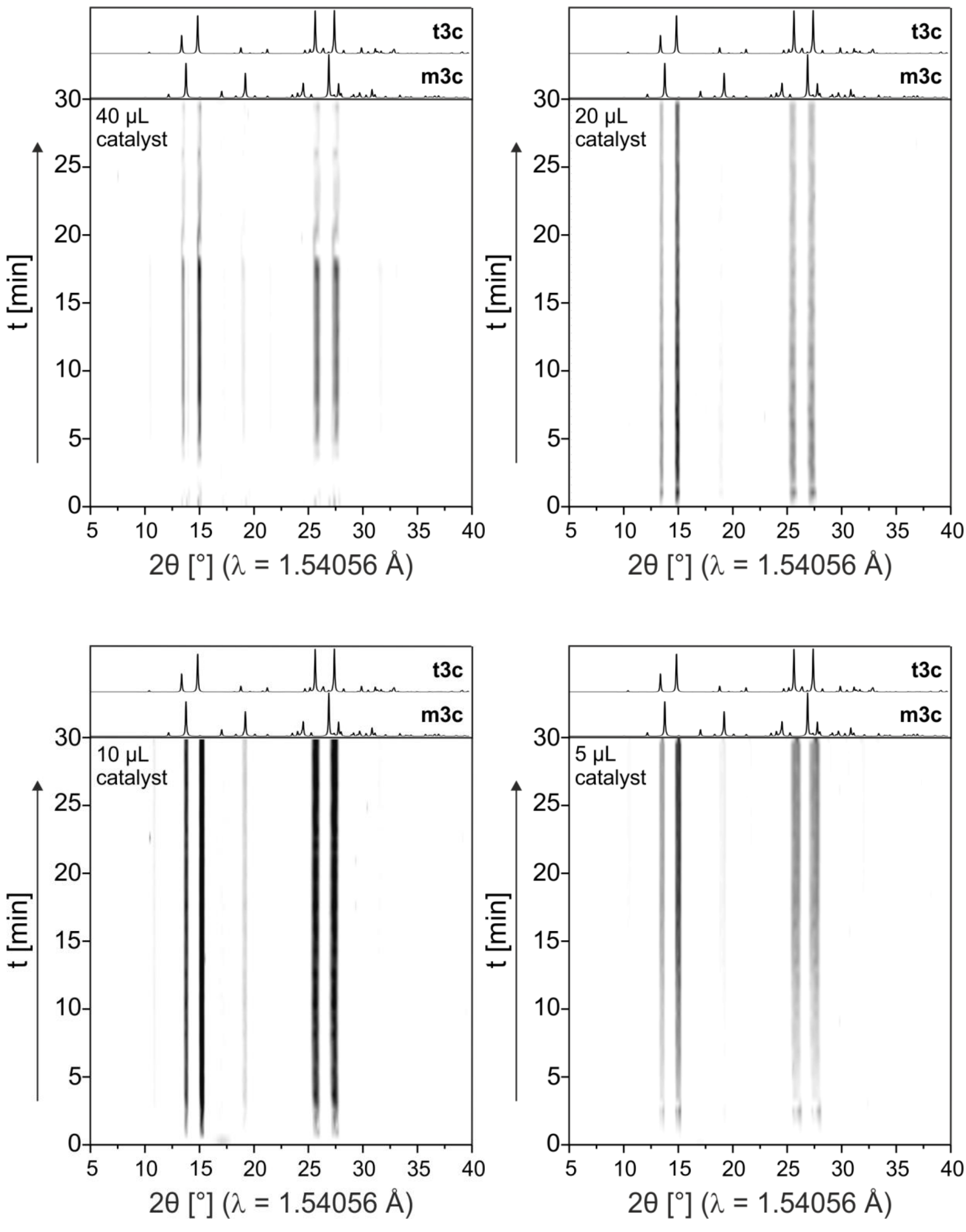

Figure S1: Time-resolved in situ XRPD pattern of the mechanochemical Knoevenagel condensation of **1a** and **2**. The amount of catalyst can be read in each top left corner.

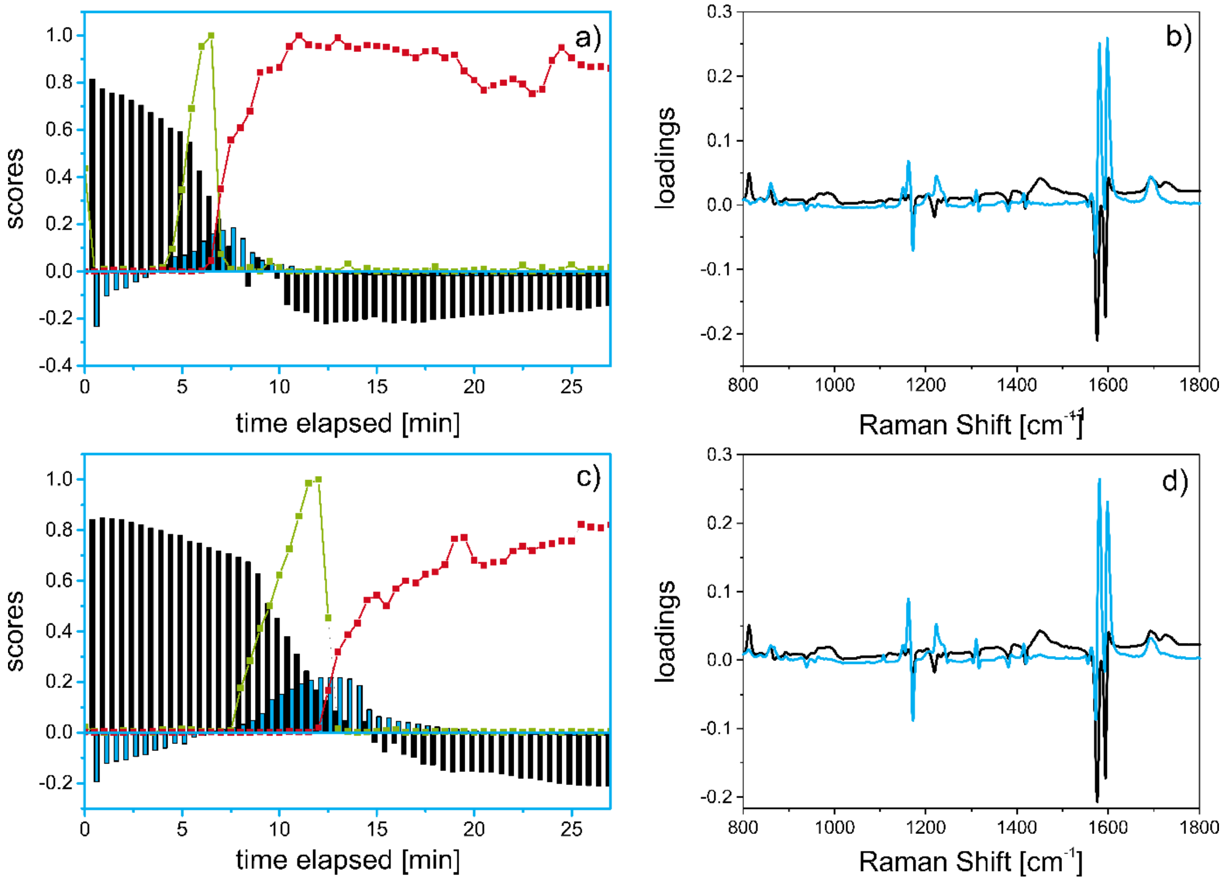

Figure S2: PCA of Raman spectra for the first 27 minutes of the reaction: a,b) Scores and loadings plots of the first (black) and second principal component (blue) for 50 Hz milling frequency, c,d) Scores and loadings plots of PC1 (black) and PC2 (blue) for 30 Hz milling frequency. For comparison, XRPD data are displayed with scatter angles 2θ = 13,87 (green) and 14,78° (red) for the monoclinic and triclinic polymorph of fluorobenzylidene malononitrile (**3a**).

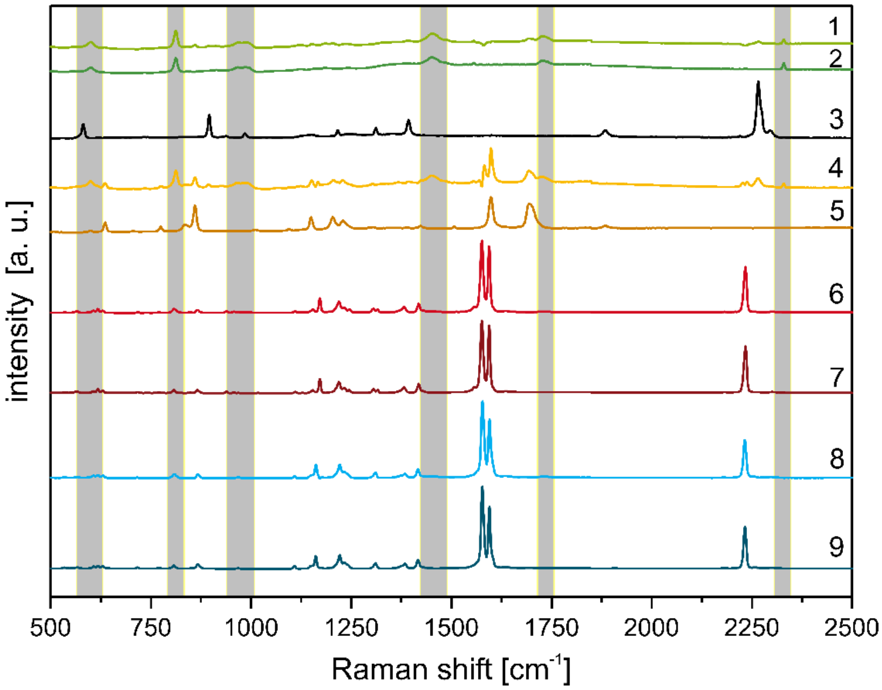

Figure S3: Comparison of the component spectra from MCR of the reaction with 30 Hz milling frequency (1, 4, 6, 8) and the Raman spectra of PMMA (2), malononitrile (3), fluorinated benzaldehyde (5), triclinic (7) and monoclinic fluorobenzylidene malonitrile (9). The spectra are sorted by spectral similarity for better identification. Shaded areas indicate the spectral contribution of PMMA contained in all spectra obtained by MCR.

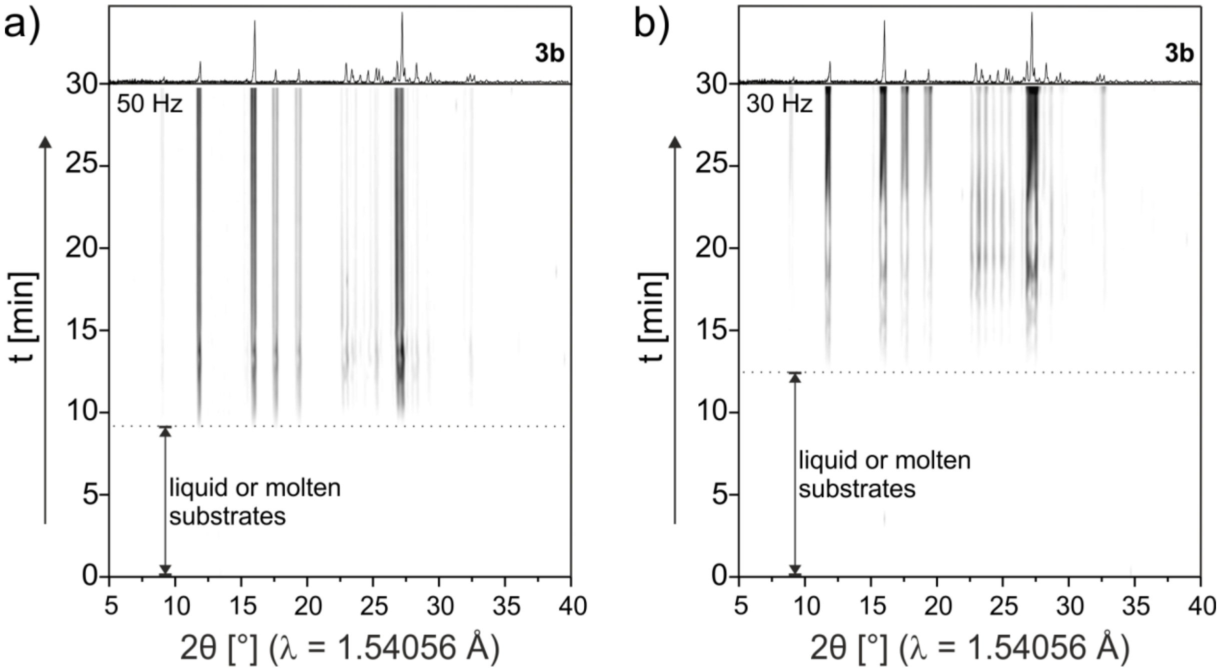

Figure S4: Time-resolved in situ XRPD pattern of the mechanochemical Knoevenagel condensation of **1b** and **2** at a) 50 Hz and b) 30 Hz.

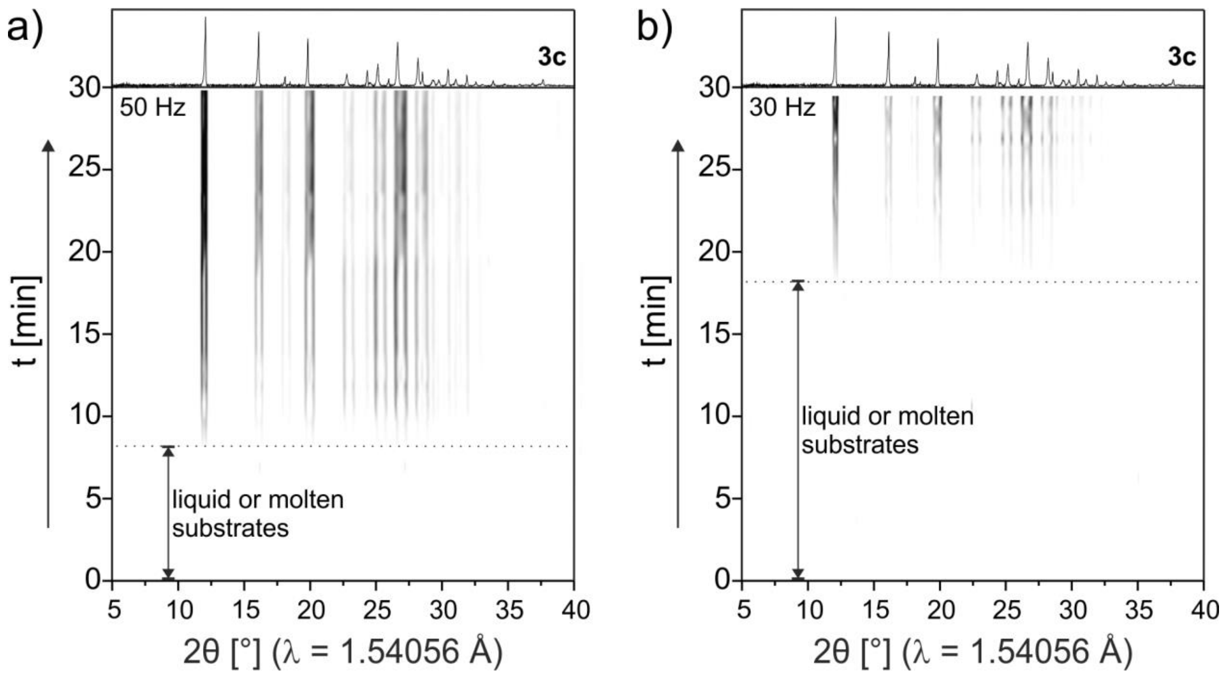

Figure S5: Time-resolved in situ XRPD pattern of the mechanochemical Knoevenagel condensation of **1c** and **2** at a) 50 Hz and b) 30 Hz.
